# Trajectories of anxiety and health related quality of life during pregnancy

**DOI:** 10.1371/journal.pone.0181149

**Published:** 2017-07-24

**Authors:** K. Oliver Schubert, Tracy Air, Scott R. Clark, Luke E. Grzeskowiak, Edward Miller, Gustaaf A. Dekker, Bernhard T. Baune, Vicki L. Clifton

**Affiliations:** 1 Discipline of Psychiatry, The University of Adelaide, Adelaide, Australia; 2 SA Health, Northern Adelaide Local Health Network, Mental Health Services, Elizabeth Vale, Australia; 3 Robinson Research Institute, School of Medicine, University of Adelaide, Adelaide, Australia; 4 SA Pharmacy, Flinders Medical Centre, Adelaide, Australia; 5 Mater Medical Research Institute, University of Queensland, Brisbane, Australia; Universita Cattolica del Sacro Cuore Sede di Roma, ITALY

## Abstract

Anxiety and health related Quality of Life (HRQoL) have emerged as important mental health measures in obstetric care. Few studies have systematically examined the longitudinal trajectories of anxiety and HRQoL in pregnancy. Using a linear growth modeling strategy, we analyzed the course of State-Trait Anxiety Inventory (STAI)- and Short Form (36) Health Survey (SF-36) scores between the 12^th^ and the 36^th^ week of gestation, in a sample of 355 women. We additionally analyzed the impact of depressive symptoms and a chronic medical condition (asthma), on STAI and SF-36 trajectory curves. STAI scores remained stable throughout pregnancy. A previous history of anxiety increased the overall STAI scores. Asthma and depressive symptoms scores had no impact on the STAI trajectory. Physical SF-36 scores decreased over the course of pregnancy, whereas mental SF-36 trended towards improvement. Asthma reduced physical SF-36 overall. While high depressive symptoms decreased the overall mental SF-36, they were also significantly associated with mental SF-36 improvements over time. Anxiety symptoms are stable during pregnancy and are not modulated by depressive symptoms or asthma. Physical HRQoL declines in pregnancy. In contrast, mental HRQoL appears to improve, particularly in women with high initial levels of depressive symptoms.

## Introduction

The epidemiology and the impact of affective disorders such as major depressive disorder (MDD) or generalized anxiety Disorder (GAD) in pregnancy have been studied extensively [1]. Less information exists about the longitudinal course of mental health measures in pregnant women. Recent evidence suggests that longitudinally elevated levels of mental discomfort, even in the absence of a categorically diagnosable psychiatric disorder, could be important.

The exploration of longitudinal trajectories of psychological and physical symptoms of interest, as opposed to cross-sectional sampling, has proliferated in the biomedical literature over the recent years. There is increasing recognition that the systematic measurement of health parameters over time can reveal novel information about aetiological trajectory drivers [2, 3], which in turn have the potential to inform preventive strategies and to further improve the efficacy of pre-natal screening programs.

Anxiety symptoms during pregnancy have emerged as an independent risk factor for adverse obstetric and developmental outcomes. Anxiety levels are closely linked to maternal cortisol trajectories [4]. Children of mothers with high levels of anxiety during pregnancy are at risk of displaying impaired fetal growth patterns [5], particularly in males [6]. Elevated anxiety symptoms during pregnancy, together with depressive symptoms and stress, predict long term behavioural and emotional problems in the offspring that can be detected as late as adolescence and adulthood [7, 8]. In mothers, prenatal anxiety has also been implicated as a potential independent risk factor for the development of postpartum depression [9].

Health related Quality of Life (HRQoL), a concept of subjectively experienced wellbeing measured by standardized psychological assessment tools, is a health outcome measure that is widely used to evaluate clinical interventions and to guide clinical management [10]. HRQoL measures, which assess patient’s views of the impact of a given condition across a variety of areas of daily living and functioning, are highly relevant to pregnant women [11]. Previous longitudinal studies have suggested that HRQoL declines during pregnancy [12], and that health problems such as depressive symptoms are associated with lower HRQoL ratings by pregnant women [13].

Pregnant women with pre-existing and mental- and physical health difficulties are thought to be at increased risk for obstetric complications and poorer birth outcomes. Therefore, prevention and early recognition of adverse mental health outcomes form part of routine care in obstetric practice. Asthma and major depressive disorder (MDD), for example, are common health conditions that are routinely screened for in obstetric settings, with the goal of achieving optimal treatment to minimize their role as recognized risk factors for adverse obstetric outcomes [14–18]. For example, the presence of maternal asthma is associated with poorer perinatal outcomes, including increased risk of low birth weight, small-for-gestational age and preterm birth. In addition, mothers with asthma are at greater risk of developing pre-eclampsia, gestational diabetes and Caesarean section [19, 20]. Similarly, MDD during pregnancy is associated with the risk for preterm birth and low birth weight [21, 22].

**Fig 1:**
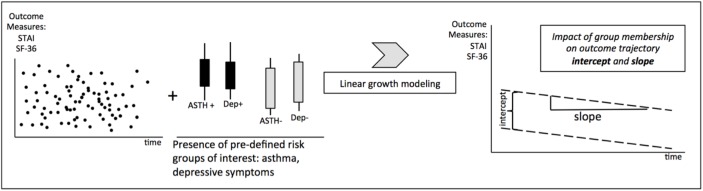
Experimental design. We used longitudinally assessed measures of anxiety (STAI) and HRQoL (SF-36) for our analysis. The analytic strategy was hypothesis-driven and defined experimental groups of interest a priori. Pre-defined experimental groups included “presence of asthma” (AST +) versus “no asthma” (AST-) and “presence of depression” (Dep+) versus “no depression” (dep-). The impact of these dichotomies on the overall longitudinal level of outcome variables (“intercept”) and on their rate of change over time (“slope”) was assessed. Statistically, we used a linear growth modeling strategy.

As far as we are aware, no previous studies have systematically investigated the longitudinal course of anxiety symptoms and HRQoL measures during pregnancy, and little is known about the modulators of their trajectories. In this study, we analysed the longitudinal trajectories of anxiety symptoms and HRQoL scores in a cohort of 355 pregnant women recruited from a socioeconomically disadvantaged area in Adelaide, South Australia. Using a linear growth modeling strategy to examine the pregnancy trajectories of anxiety and HRQoL, we hypothesized that the presence of a chronic medical condition (asthma) and/or high levels of depressive symptoms at an early pregnancy stage would negatively impact on overall anxiety and HRQoL levels (“intercepts”), as well as on their trajectory throughout the pregnancy (“slopes”)(Fig 1).

## Methods

Approval for the study was provided by The Queen Elizabeth Hospital, Lyell McEwin Hospital, and Modbury Hospital Human Research Ethics Committee and The University of Adelaide Human Research Ethics Committee. All women gave written informed consent.

### Prospective study of pregnant women with and without asthma

Participants for this study were recruited in the context of a non-interventional prospective cohort study evaluating the impact maternal asthma on perinatal outcomes. Mental health measures, as specifically analyzed in the current study, were collected as secondary outcomes of this larger cohort. Pregnant women with and without asthma were recruited through the Lyell McEwin Hospital antenatal clinics, a public health service in South Australia, Australia, between May 2009 and May 2012. Women were assessed by a midwife, with additional respiratory training, at 12, 20, 28, and 36 weeks gestation. The midwife utilised a standardised data collection tool to collect demographic data including maternal weight, height, previous obstetric history, medical history. Maternal smoking was assessed by maternal self-report, with women classified according to smoking status during the first antenatal visit. Socioeconomic status for each woman was determined using her residential postcode at the time of delivery. Women were then ranked according to their level of advantage, or relative disadvantage, based on data from the Socio-Economic Indexes for Areas, calculated from the Australian Bureau of Statistics’ 5-yearly Census of Population and Housing. These indexes are widely used measures of relative socioeconomic status. Among women with asthma, additional data were collected related to asthma control, medication use, lung function, which are described in more detail in previous publications [23, 24]. The same midwife saw women for each of their respective visits. Patients assessed as unstable and requiring medical review were referred to their primary care physician or to a respiratory physician.

Data was prospectively collected at each study visit.

### Assessment of maternal anxiety symptoms, health-related quality of life (HRQoL), and depressive symptoms

Symptoms of anxiety and HRQoL were assessed longitudinally at 12, 20, 28, and 36 weeks gestation. Anxiety symptoms were assessed using the State-Trait Anxiety Inventory (STAI)[25]. 20 items assess trait anxiety and state anxiety, respectively. State anxiety items include statements such as “I am tense; I am worried”, or “I feel calm; I feel secure.” Trait anxiety items include statements such as “I worry too much over something that really doesn’t matter”, or “I am content; I am a steady person.” All items are rated on a 4-point scale (e.g., from “Almost Never” to “Almost Always”). Higher total scores indicate greater anxiety. HRQoL was assessed using the Short Form (36) Health Survey (SF-36)[26]. The SF-36 assesses the impact of physical or emotional problems on a variety of health concepts, including physical activities, social activities, bodily pain, general mental health (psychological distress and well-being), vitality (energy and fatigue), and general health perceptions. The standard form of the instruments asks for participants to reply to questions according to how they have felt over the previous week. The items use Likert-type scales, some with 5 or 6 points and others with 2 or 3 points. Two summary scores are produced: the Mental Health Component Score and the Physical Health Component Score.

Depressive symptom severity at 12 weeks gestation was assessed using Edinburgh Postnatal Depression Score Questionnaire (EPDS)[27]. The EPDS is a set of 10 screening questions to assess whether women have symptoms that are common with depression and anxiety during pregnancy and in the year following the birth of a child. The ‘high EPDS’ group was defined by an EPDS score of 13 or more points on the overall 30-point scale. A previous history of maternal depression, including a previous history of postnatal depression (PND), anxiety, and family history of mental ill health were identified based on maternal self-report. For details on previous maternal mental health problems and previous treatments, and for screening for previous emotional, physical, or sexual abuse, the Antenatal Risk Questionnaire (ANRQ)[28] was used.

### Statistical methods

All data were analysed using Stata 12.0. The data were inspected graphically to establish normality prior to analysis. To identify potential confounders, we used chi squares or Student’s t-test for testing the association between demographic, psychiatric or medical characteristics of the sample at baseline and high depressive symptom status as identified by the EPDS.

For multivariable analysis, linear growth models of the State-Trait Total score and the SF-36 Mental and Physical subscales were performed against positive screening for depressive symptoms at baseline (as per the EPDS) and asthma, adjusting for a history of anxiety, a history of PND, current antidepressant usage, a history of physical and/or sexual abuse, parity and a family history of psychiatric disorder. Subjects with missing data were included in analyses, as data collection at every time point does not need to be adhered to in growth models.

## Results

### Sample characteristics

Table 1 displays the baseline characteristics of the sample of n = 355 participants included in the analysis. Participants were on average 26.2±5.3 years old, and 193 (54%) had a diagnosis of asthma. 77 women (22%) of the sample reported that they were current smokers. 39 women reported a previous diagnosis of postnatal depression (PND), and 136 (38%) said they had a history of any anxiety disorder. Average baseline EPDQ, STAI, and SF-36 scores for the entire sample were in the low (i.e. non-pathological) range for each of these assessment tools.

**Table 1:** Sample characteristics.

	Entire sample (n = 355)
Age (years)	26.2±5.3
Diagnosis of Asthma	193 (54%)
Gravida	2.6±1.7
Parity	0.94±1.1
Current Smoker	77 (22%)
Married	251 (71%)
History of Anxiety	136 (38%)
History of PND	39 (11%)
Baseline EPDS	5.4±4.8
Baseline mean STAI	29.2±10.6
Baseline mean SF-36 Mental	46.7±8.5
Baseline mean SF-36 Physical	42.6±7.0

Demographics and clinical characteristics of the entire sample of 355 pregnant women. PND: Postnatal Depression; EPDS: Edinbourgh Postnatal Depression Scale; STAI: State Trait Anxiety Inventory; SF-36: Short Form (36) Health Survey.

### Characteristics of women with low levels of depressive symptoms (low EPDS group) versus women with higher levels of depressive symptoms (high EPDS group)

Table 2 shows comparisons between the experimental groups low and high levels of depressive symptoms. Significantly more of the High EPDS group had asthma (X2 = 4.6, df = 1, p = 0.03), a history of anxiety (X2 = 25.2, df = 1, p<0.001), a history of PND (X2 = 22.8, df = 1, p<0.001), and a family history of psychiatric disorder (X2 = 6.5, df = 1, p = 0.04). They also were significantly more likely to have experienced physical and/or sexual abuse (X2 = 17.2, df = 1, p<0.001), and were more likely prescribed anti-depressants (X2 = 29.2, df = 1, p<0.001).

**Table 2:** Characteristics of the Low EPDS and High EPDS groups.

	Low EPDS (n = 323)	High EPDS (n = 32)	p
Age (years)	26.3±5.3	25.1±5.1	0.25
Diagnosis of Asthma	171 (53%)	22 (73%)	0.03
Gravida	2.5±1.7	2.6±1.4	0.72
Parity	0.95±1.1	0.88±0.9	0.70
History of Anxiety	111 (33%)	25 (78%)	<0.001
History of PND	28 (8%)	11 (34%)	<0.001
Antidepressant use at 12 weeks gestation	17 (5%)	10 (31%)	<0.001
Family History of Psychiatric Disorder	86 (26%)	14 (44%)	0.02
Reported Physical/Sexual Abuse	55 (17%)	14 (44%)	0.001

Demographic and clinical characteristics of the study population, divided into participants who scored low (<13 points) on the Edinburgh Postnatal Depression Scale (EPDS), versus those who reported high scores (>13 points). Factors that were significantly different between groups were considered as co-variants in the mixed model analyses. PND: Postnatal Depression.

### Characteristics of women with a diagnosis of asthma

Table 3 describes the characteristics of the participant group diagnosed with asthma, compared to participants without asthma. Women with an asthma history were more likely to report a history of anxiety (46% vs 24%, p<0.0001), had a higher baseline EPDS score (6.1±5.1 vs 4.4±3.9, p<0.001), and higher STAI scores (30.6±11.3 vs 27.5±9.5, p<0.05). Additionally, women with an asthma history reported poorer HRQoL, both on the SF-36 physical (40.9±7.4 vs 44.6±6.0, p<0.001) and SF-36 mental (45.3±9.3 vs 48.3±7.6, p<0.01) subscales.

**Table 3:** Characteristics of Asthma and No Asthma groups.

	Asthma (n = 193)	No Asthma (n = 159)	p
Age	25.9±5.4	26.5±5.2	0.33
Gravida	2.5±1.7	2.5±1.7	0.97
Parity	0.9±1.0	1.0±1.2	0.56
History of Anxiety	90 (46%)	38 (24%)	<0.001
History of PND	24 (12%)	12 (8%)	0.12
BMI	28.7±7.2	27.7±7.1	0.16
Smoker	43 (22%)	34 (21%)	0.94
Any ICS	96	NA	NA
Baseline EDPD	6.1±5.1	4.4±3.9	<0.001
Baseline STAI	30.6±11.3	27.5±9.5	<0.05
Baseline SF-36 Physical	40.9±7.4	44.6±6.0	<0.001
Baseline SF-36 Mental	45.3±9.3	48.3±7.6	<0.01

Demographic and clinical characteristics of the study population, divided into participants with and without a history of asthma. PND: Postnatal Depression; BMI: body mass index; EDPS: Edinburgh Postnatal Depression Scale; STAI: State Trait Anxiety Questionnaire. SF-36: Short Form (36) Health Survey. NS: non significant at p<0.05; NA: not applicable.

### Analysis of the longitudinal trajectory of STAI scores throughout pregnancy

**Fig 2:**
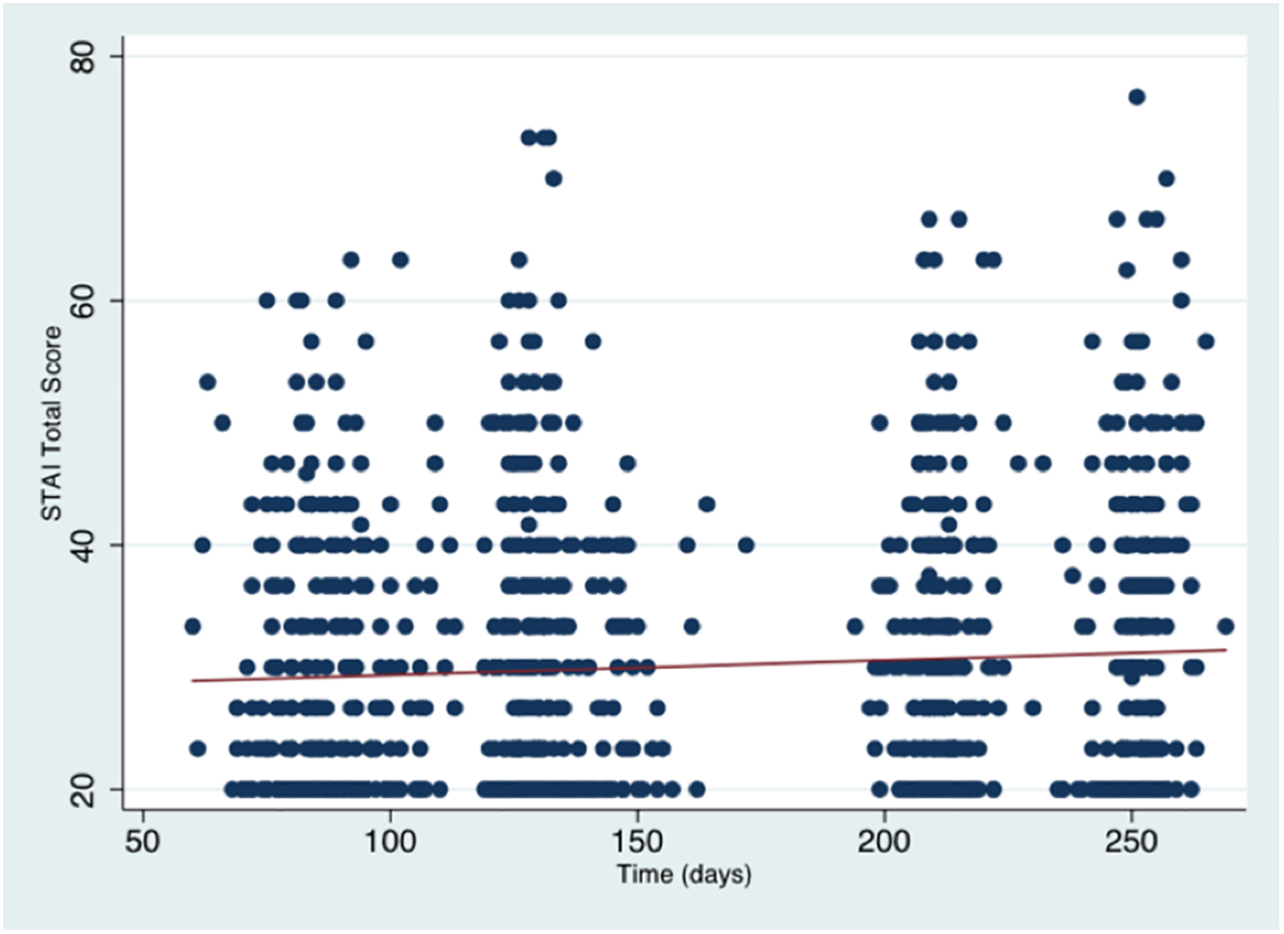
Scatterplot of all participants’ STAI scores throughout pregnancy. The red line indicates the regression of mean scores over time. Statistically, STAI scores were stable over time for the overall cohort (β = 0.01, p = 0.18).

Average STAI scores remained statistically stable over time throughout pregnancy (β = 0.01, p = 0.18), and the majority of women in the sample reported stable low levels of anxiety symptoms throughout pregnancy (Fig 2, Table 4) [29, 30]

**Table 4:** Linear growth model coefficients for longitudinal change on State-Trait Anxiety Inventory (STAI) scores.

	β-coefficient estimate	Standard Error	p-value
***Effects on overall STAI***			
High EPDS	-0.79	6.1	0.90
Diagnosis of Asthma	1.41	1.7	0.41
Time (days)	0.01	0.01	0.18
Current antidepressant use	1.17	2.1	0.46
History of PND	-1.34	1.8	0.31
History of Anxiety	2.46	1.1	0.03
Family History of Psychiatric Disorder	0.09	0.05	0.08
History of Physical/Sexual Abuse	2.20	1.2	0.07
Parity	-0.93	0.1	0.35
***Interaction effects***			
EPDS x Time	0.02	0.03	0.51
Asthma x Time	0.001	0.01	0.96
Asthma x EPDS	9.49	7.4	0.12
Asthma x EPDS x Time	-0.02	0.04	0.65

The upper section of Table 4 describes the effects of clinical parameters on overall State-Trait Anxiety Inventory (STAI) scores (intercept). The lower section of the table describes the longitudinal interaction effects of time, baseline Edinburgh Postnatal Depression Scale (EPDS) score, and asthma on the STAI trajectory.

Linear growth model analysis of of participants’ STAI trajectories (Table 4) revealed that women with a history of anxiety reported overall significantly higher scores on the STAI (β = 2.45, p = 0.03). There were no significant interaction effects between STAI and the EPDS and asthma groups over time (Table 4), indicating that the presence of depression and/or asthma did not moderate the longitudinal course of anxiety symptoms.

### Analysis of the longitudinal trajectory of SF-36 physical and mental subscales

#### SF-36 physical subscale

**Fig 3:**
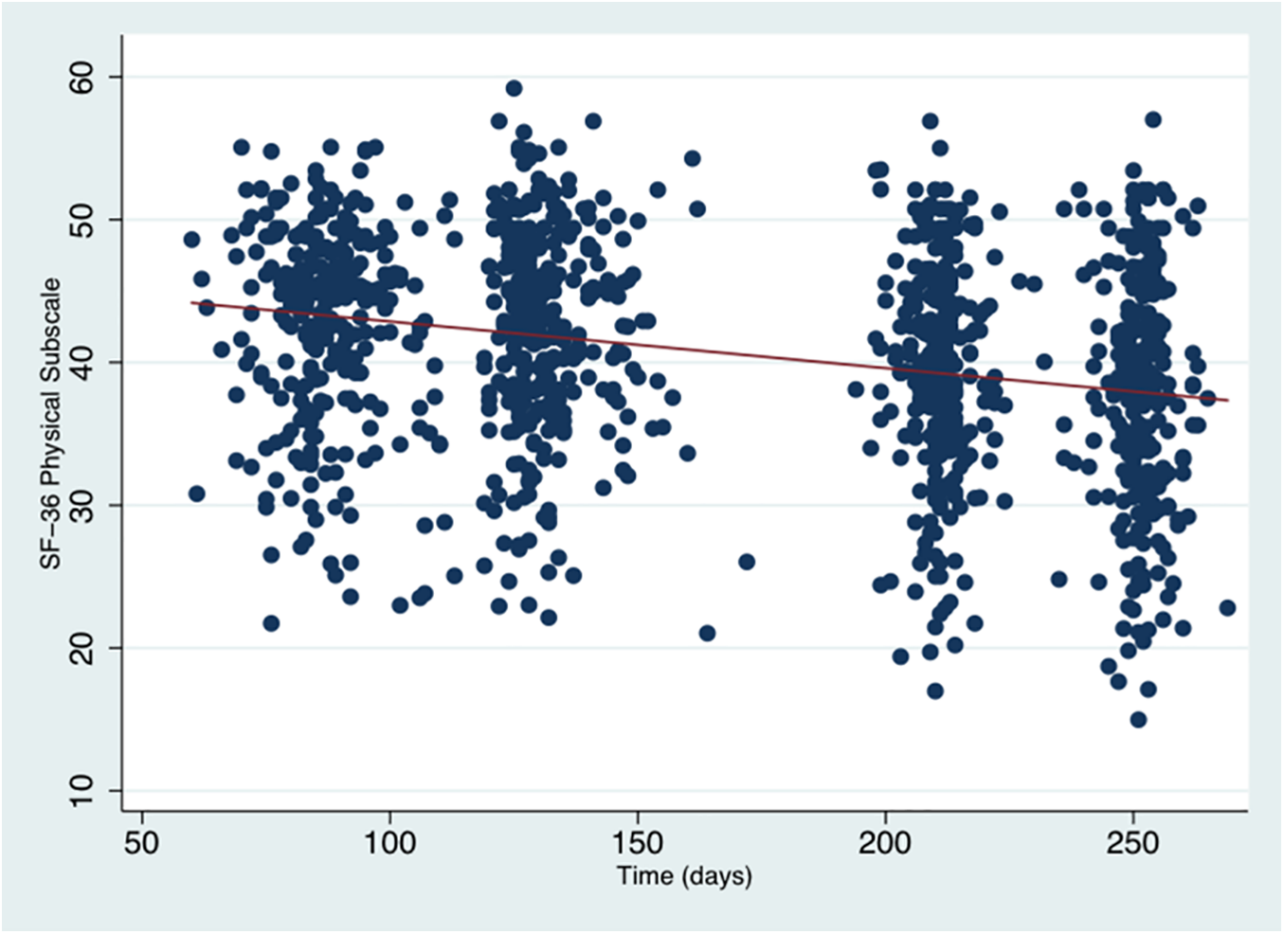
Scatterplot of SF-36 physical subscale scores in all participants across pregnancy. The red line indicates the regression of mean scores over time. Overall, SF-36 physical scores declined over the course of pregnancy (β = -0.04, p<0.001).

For the entire sample, scores on the physical subscale significantly decreased over time (β = -0.04, p<0.001), indicating a reduction in subjective physical wellbeing (Fig 3, Table 5).

**Table 5:** Linear growth model coefficients for longitudinal change on the SF-36 physical subscale.

	β-coefficient estimate	Standard Error	p-value
***Effects on overall SF-36 Physical Scale***			
High EPDS	-0.69	4.4	0.88
Asthma	-3.95	1.2	0.001
Time (days)	-0.04	0.01	< 0.001
Antidepressants	-0.14	1.4	0.99
History of Diabetes	-3.13	2.0	0.12
History of Anxiety	-0.92	0.8	0.22
History of Physical/Sexual Abuse	-1.65	0.8	0.07
Parity	-0.43	0.66	0.52
***Interaction effects***			
EPDS x Time	0.01	0.03	0.71
Asthma x Time	0.01	0.01	0.12
Asthma x EPDS	5.16	5.1	0.32
Asthma x EPDS x Time	-0.03	0.03	0.26

The upper section of Table 5 describes the effects of clinical parameters on overall SF-36 Physical Subscale scores (intercept). The lower section of the table describes the longitudinal interaction effects of time, baseline Edinburgh Postnatal Depression Scale (EPDS) score, and asthma on the SF-36 Physical trajectory.

Linear growth model analysis of the participants’ SF-36 physical trajectories indicated that the asthma group performed significantly worse compared to others on the SF-36 physical health subscale (intercept; β = -3.95, p<0.001). There were no significant interaction effects between Physical SF-3 and the EPDS and Asthma groups over time (Table 5).

#### SF-36 mental subscale

**Fig 4:**
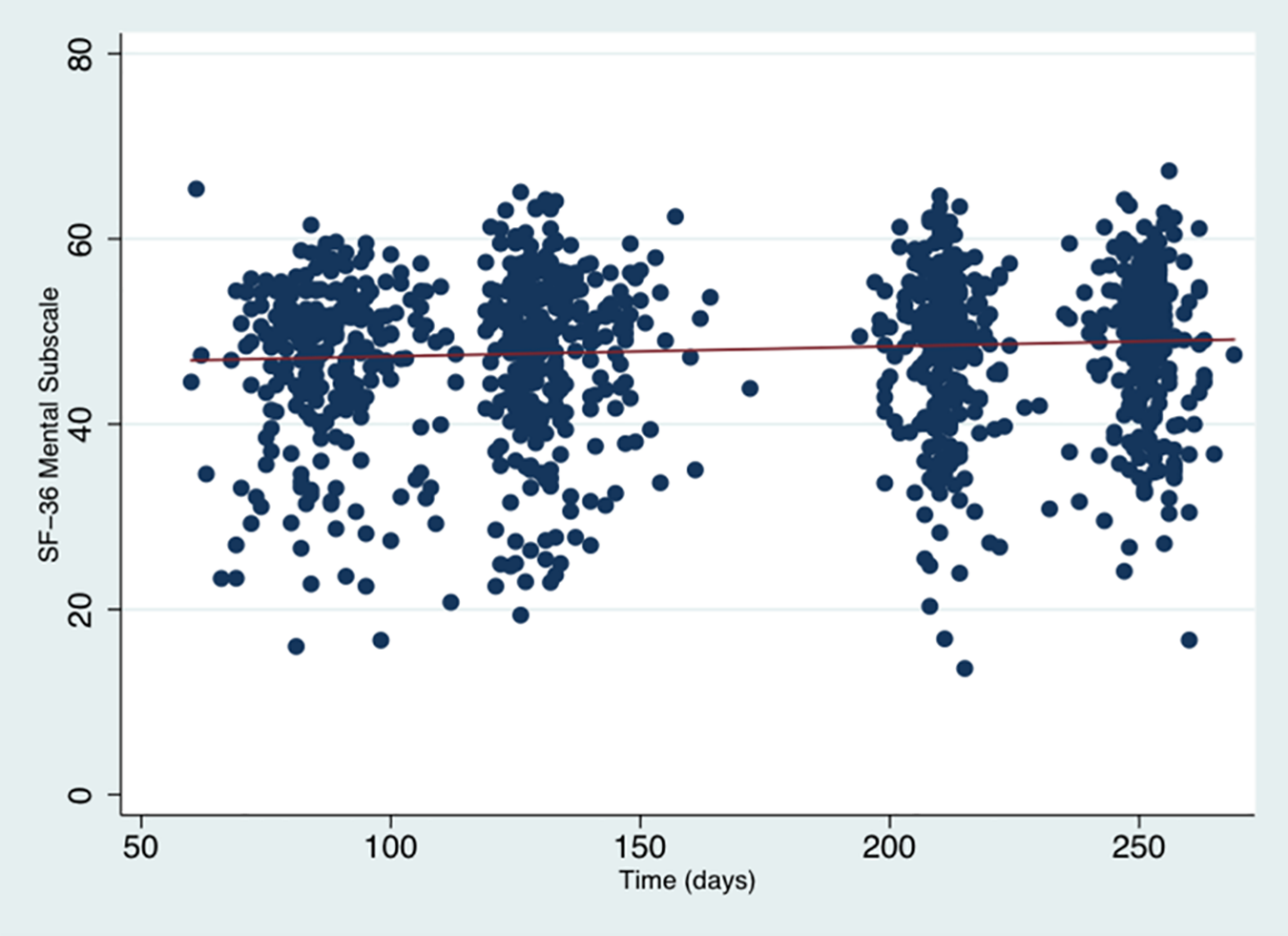
Scatter plot of SF-36 mental subscale scores in all participants across pregnancy. The red line indicates the regression of mean scores over time. We detected a non-significant trend towards subtle improvement of SF-36 mental subscale scores over the course of pregnancy (β = 0.01, p = 0.06).

SF-36 mental subscale levels for the entire sample increased during the course of the pregnancy in the entire cohort, but not significantly so (β = 0.01, p = 0.06) (Fig 4, Table 6), indicating possible subtle improvements in perceived mental wellbeing over the course of pregnancy.

**Table 6:** Linear growth model coefficients for longitudinal change on the SF-36 mental subscale.

	β-coefficient estimate	Standard Error	p-value
***Overall effect on SF-36 Mental Scale***			
High EPDS	-16.89	4.8	<0.001
Asthma	-1.33	1.3	0.33
Time (days)	0.01	0.01	0.06
Antidepressants	-0.09	1.5	0.95
History of Anxiety	-1.82	0.8	0.02
History of PND	-2.42	1.3	0.07
History of Stress	-2.13	0.8	0.006
History of Emotional Problems	-2.61	1.1	0.01
Problems with Accommodation	-2.85	1.5	0.06
History of Emotional Abuse	1.32	1.3	0.32
History of Physical/Sexual Abuse	-1.54	1.0	0.14
Parity	0.95	0.69	0.17
***Interaction effects***			
EPDS x Time	0.08	0.03	0.007
Asthma x Time	0.001	0.01	0.83
Asthma x EPDS	7.52	5.8	0.20
Asthma x EPDS x Time	-0.06	0.03	0.06

The upper section of Table 6 describes the effects of clinical parameters on overall SF-36 Mental Subscale scores (intercept). The lower section of the table describes the longitudinal interaction effects of time, baseline Edinburgh Postnatal Depression Scale (EPDS) score, and asthma on the SF-36 Mental trajectory.

Linear growth model analysis revealed that participants with a history of anxiety, a history of stress, a history of emotional problems and issues with accommodation reported significantly lower initial scores on the SF-36 Mental Subscale (Table 6). There was a significant EPDS x Time interaction effect (β = 0.08, p = 0.007). The high EPDS group initially began with lower scores on the SF-36 Mental subscale (indicating poorer subjective experience of mental health), but improved at a faster rate than the low EPDS group (Table 6).

## Discussion

We carried out linear growth model analyses to describe the longitudinal course of anxiety symptoms and HRQoL between the 12^th^ and the 36^th^ week of pregnancy. Further, we investigated the impact of asthma and depressive symptoms at baseline on anxiety and HRQoL trajectories. In a cohort of 355 pregnant women, recruited from a socioeconomically disadvantaged area in South Australia, we firstly found that anxiety symptoms are relatively stable throughout pregnancy. A previous history of anxiety was the only factor that increased the overall longitudinal level of anxiety symptoms (intercept). Asthma or depressive symptoms at baseline had no impact on the rate of symptom change over time (slope). Secondly, we found that physical HRQoL significantly decreased over the course of pregnancy, whereas mental HRQoL trended towards improvement. As expected, asthma reduced physical HRQoL overall (intercept), but had no impact on the rate of deceleration over time (slope). Surprisingly, while high EPDS status decreased mental HRQoL overall (intercept), it was also significantly associated with faster mental HRQoL improvements over time compared to the low EPDS group.

The course of anxiety symptoms throughout pregnancy has previously been described in a number of cross-sectional studies, albeit not by specific trajectory analysis [9, 31, 32]. These studies report relatively high levels of anxiety during the first trimester, then a decrease in the 2^nd^ trimester followed by increasing symptoms during the 3^nd^ trimester up to childbirth. Such a possible U-shaped pattern was not borne out in our analysis, which suggests overall stable anxiety trajectories in our sample. Our finding that higher levels of anxiety symptoms at the beginning of pregnancy significantly predict higher symptom levels throughout the rest of pregnancy is consistent with previous reports [9, 32].

The STAI has previously been employed as an assessment tool in pregnancy, and has been used in several studies to measure the impact of psychological treatments delivered to pregnant women (for review see [33]). Compared to non-pregnant populations, STAI scores are estimated to be elevated in pregnancy, and in many reports reach mean STAI values around 40 [33]. Overall anxiety levels in our sample were comparably modest around average STAI scores of about 30, a discrepancy that could be explained by potential recruitment bias in these previous investigations aiming at therapy effects. Nevertheless, longitudinally elevated and clinically relevant anxiety levels [34] affected a considerable proportion of women in our sample (Fig 2).

Contrary to our hypotheses, neither asthma nor high EPDS status altered the intercept or slope of anxiety trajectories in pregnancy. No previous study has applied linear growth modeling to longitudinal symptom courses in pregnant women, to delineate this interaction between anxiety symptoms with mood or physical illness. Our findings highlight that anxiety changes over time could be independent of these factors. This underscores the importance of specifically assessing and treating anxiety in prenatal care [35]. Currently, such specific anxiety screens are not routinely carried out across primary care and obstetric settings [36].

Our finding on physical HRQoL was consistent with our prior expectations. Similar to previous studies, we found that physical SF-36 scores decreased throughout pregnancy [37, 38], and asthma status further lowered subjective mental wellbeing. These decreases of physical well-being could be linked to increasing pain and discomfort, and accompanying restrictions in role functioning.

Mental SF-36 scores trended towards improvement over time, again consistent with previous reports [38]. As expected, high levels of baseline depressive symptoms lowered the overall level of mental wellbeing, similar to the findings of a previous cross-sectional study [39]. However, women with high baseline EPDS scores experienced greater improvements in mental wellbeing than women with low initial depression scores. This finding suggests that advancing pregnancy may, at least in part, counteract the negative effects of depression on women’s perceived mental wellbeing over time. Another question is whether the observed improvement in mental SF-36 in depressed women is predominantly driven by improvements in depressive symptoms. Previously published trajectory analyses of the course of depressive symptoms during pregnancy suggest otherwise; the 3 trajectory groups described by a latent growth modeling study of Beck’s Depression Inventory (BDI) scores exhibited stable or increasing symptoms over time, but no group with decreasing depressive symptoms [40]. Hence, it is likely that factors other than depressive symptoms contribute to improving mental wellbeing in late pregnancy. It is also notable that asthma did not significantly alter trajectories of mental SF-36 scores. A previous study analyzing the effect of chronic rheumatic illnesses on HRQoL in pregnancy came to similar conclusions, and additionally reported no impact of acute illness exacerbations on subjective mental wellbeing [41].

### Limitations

This study relied on self-reports for assessment of anxiety, HRQoL, and depressive symptoms. Whilst all assessment tools are evidence-based in terms of their validity, there is ultimately no certainty about reported symptom levels, and it is possible that clinician-rated scales would have led to different conclusions. Further, it is possible that a previous history of specific anxiety disorder such as panic disorder, social phobia, or generalized anxiety disorder (GAD) is associated with unique STAI trajectories in pregnancy. We did not specifically assess participants for these disorders using validated structured clinical interviews, and therefore cannot comment on this issue. Future research should consider pre-existing DSM-defined anxiety disorders when carrying out trajectory analyses of anxiety symptoms.

The study was conducted in a socioeconomically disadvantaged area of South Australia, and study participants had lower levels of education, lower employment rates, and higher rates of psychosocial adversity, including a history of abuse, than would be expected in a representative community sample in Australia. Therefore, it is possible that our findings are not generalizable to the entire population. Future research should include ‘control’ populations where the above factors play less of a role.

## Conclusions

The linear growth model analyses of this study suggest that anxiety symptoms are relatively stable throughout pregnancy, whereas physical HRQoL decreases and mental HRQoL improves. Our findings indicate that chronic health conditions and depressive symptoms in early pregnancy have no impact on the trajectory characteristics of anxiety. In contrast, these factors modulate physical and mental HRQoL. Interestingly, we found that advancing pregnancy may improve subjective mental wellbeing in women who struggle with high levels of depressive symptoms in the first trimester.

Given the complex interactions between anxiety, quality of life, depressive symptoms, and physical health, our study highlights the importance of comprehensive assessments of mental health and subjective wellbeing in obstetric practice. These assessments should specifically address anxiety and HRQoL, and specific bio-psycho-social treatments should be offered to women who are reporting difficulties in these areas. Our study suggests that comprehensive perinatal mental healthcare is particularly important in socioeconomically disadvantaged populations.
